# Narrow and Deep Fano Resonances in a Rod and Concentric Square Ring-Disk Nanostructures

**DOI:** 10.3390/s130911350

**Published:** 2013-08-26

**Authors:** Yanyan Huo, Tianqing Jia, Yi Zhang, Hua Zhao, Shian Zhang, Donghai Feng, Zhenrong Sun

**Affiliations:** State Key Laboratory of Precision Spectroscopy, Department of Physics, East China Normal University, Shanghai 200062, China; E-Mails: huoyanyan2008@163.com (Y.H.); yizhang@tjpu.edu.cn (Y.Z.); zhaohuahust@163.com (H.Z.); sazhang@phy.ecnu.edu.cn (S.Z.); dhfeng@phy.ecnu.edu.cn (D.F.); zrsun@phy.ecnu.edu.cn (Z.S.)

**Keywords:** Fano resonance, plasmonic nanostructures, optical sensor, Fano linewidth spectral contrast ratio, figure of merit

## Abstract

Localized surface plasmon resonances (LSPRs) in metallic nanostructures have been studied intensely in the last decade. Fano interference is an important way to decrease the resonance linewidth and enhance the spectral detection resolution, but realizing a Fano lineshape with both a narrow linewidth and high spectral contrast-ratio is still challenging. Here we propose a metallic nanostructure consisting of a concentric square ring-disk (CSRD) nanostructure and an outside nanorod. Fano linewidth and spectral contrast ratio can be actively manipulated by adjusting the gap between the nanorod and CSRD, and by adjusting the gap between the ring and disk in CSRD. When the gap size in CSRD is reduced to 5 nm, the quadrupolar Fano linewidth is of 0.025 eV, with a contrast ratio of 80%, and the figure of merit reaches 15.

## 1. Introduction

Fano resonance in metallic nanostructures has gained much attention in recent years. It arises from the constructive and destructive interference of a narrow dark mode and a broad bright mode [1–3]. Fano resonances were considered mostly in quantum systems [4], but they have been realized in many metallic nanostructures, such as dolmen nanostructures [5,6], nanoparticle clusters [7–12], and ring-disk nanocavities [13]. The linewidth and spectral contrast ratio (CR) are the two most important factors that determine the overall performance of Fano resonances [14].

Nanorod structures are easy to fabricate, and Fano resonances are commendably realized in these nanostructures [15–19]. Zhang *et al.* proposed a plasmonic nanostructure consisting of two parallel bars and a perpendicular bar, and found deep Fano resonance in the transparency spectra [5]. Yang *et al.* reported a Fano dip in the extinction spectrum of a plasmonic nanorod dimer, which was caused by the interference between the bright mode of the short nanorod and the dark mode of the long nanorod [15]. Liu *et al.* proposed and fabricated a kind of multilayer plasmonic oligomer composed of a nanorod and two nanorod dimers, which possesses two dark quadrupole modes with energy detuning, resulting in double Fano resonances in the spectra. They demonstrated that these nanostructures can be used as three-dimensional plasmon rulers [18,19].

Plasmon resonance in a nanoring are highly tunable. The resonant frequency depends on both the diameter and the wall width [20]. Hao *et al.* reported that the antiparallel coupling of the dipolar plasmons of the disk and ring led to a sharp line-shape resonance due to less radiative losses [21]. Fu *et al.* found that higher-order Fano resonances were generated when the disk size was reduced to a certain range and they designed dual-disk ring nanostructures to manipulate the Fano resonance line shape [22,23]. Zhang *et al.* proposed a plasmonic nanostructure consisting of a nanodisk and a nanoring, which surpports multipolar resonance modes and shows both high CR and figure of merit (FOM) [24].

In recent years, rectangular rings have also attracted much attention. Kanté *et al.* reported the first experimental demonstration of an ultra-broad Fano resonance induced optical negative index band in closed rectangular metallic nanorings [25]. Cao *et al.* demonstrated a planar terahertz Fano metamaterial with an ultrahigh quality factor, which was achieved by the excitation of the nonradiative dark modes by introducing a tiny asymmetry in the split ring nanostructures [26]. Wang *et al.* investigated double Fano resonant characteristics in a planar plasmonic nanostructure by embedding a metallic nanorod in two symmetric U-shaped split ring resonators [27].

In this paper, we propose a metallic nanostructure consisting of an outside rod and a concentric square ring-disk (CSRD) nanostructure, which is abbreviated as RCSRD. Fano linewidth and CR in the RCSRD can be actively manipulated by adjusting the gap between the outside rod and CSRD, and the gap between the ring and disk in CSRD. This plasmonic nanostructure exhibits high sensing sensitivity and is easy to be fabricated by electron-beam lithography compared with above mentioned circular disk-ring nanostructures.

## 2. Plasmonic Nanostructure and Numerical Simulation

**Figure 1.**
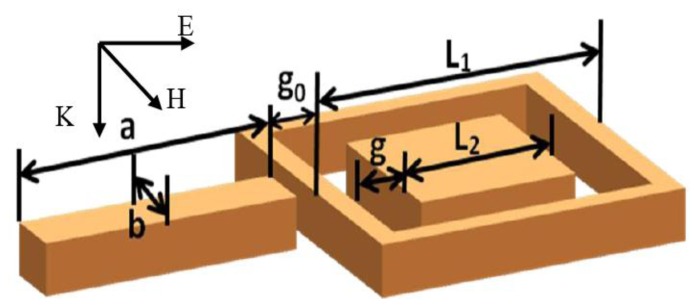
Sketch of the RCSRD and the incident light.

The RCSRD nanostructure, as shown in Figure 1, consists of an outer nanorod with length *a* and width *b*, a square nanoring with outer side length *L*_1_, and an inner square nanodisk with side length *L*_2_. The square nanodisk and the nanoring are concentric with a gap *g*. The gap width between the outside nanorod and the square nanoring is *g_0_*. In this paper, the width of outside nanorod is 60 nm, the nanoring width is 20 nm, its outer side length *L*_1_ is 240 nm, and the height of RCSRD nanostructure keeps at 60 nm. The nanostructure places in free space if without special description. A plane wave irradiates down to the RCSRD nanostructure, and the electric field is parallel to the linked line of the centers of nanorod and CSRD, as shown in Figure 1. For this polarization, this structure supports a very strong Fano resonance. If the polarization rotates by 90°, Fano resonance falls away.

The finite element method (COMSOL Multiphysics) adopting adaptive mesh is used to solve the time-harmonic three dimensional Maxwell's equations. The computation domain includes the RCSRD nanostructure, a region of free space (larger than half of the light wavelength) surrounding it, and a perfectly matched layer eliminating the reflections at the domain boundaries. The permeability of silver is *μ* = 1, with the complex permittivity sourced from [28]. A normal incident linearly polarized light source is used. The absorption spectrum of the metallic nanostructure is calculated through the volume integration of the resistive heating in the RCSRD nanostructure. The scattering spectrum is calculated by integrating the normalized electric field around a far-field transform boundary enclosing the RCSRD nanostructure. In addition, near-field information at the resonance wavelengths is directly obtained from these simulations. Surface charge plots are computed by Gauss's law, and the gradient operation is realized by implementing the up and down operators to the metal-dielectric interfaces.

## 3. Results

**Figure 2.**
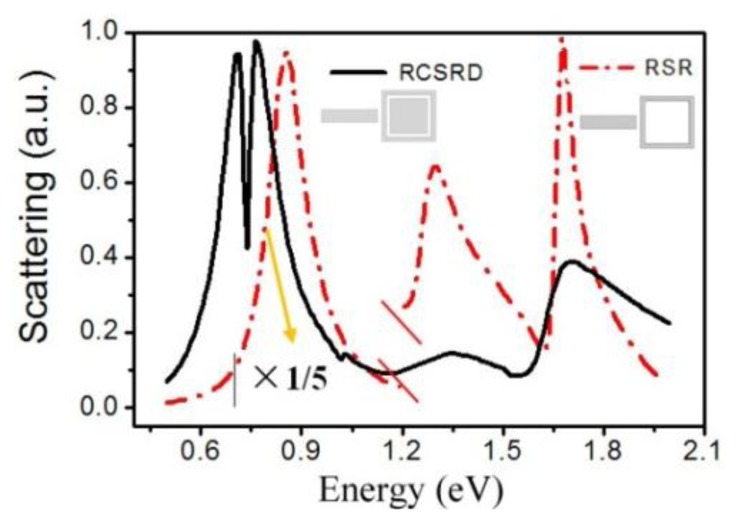
The scattering spectra of RCSRD and RSR with the same parameters of *L*_1_ = 240 nm and *g_0_* = 20 nm.

Figure 2 shows the scattering spectra of RCSRD and the rod-square ring (RSR, *i.e.*, the RCSRD nanostructure without the inner square disk). In the calculation, the nanorod length *a* and width *b* are 450 nm and 60 nm, the gap width *g* between ring and disk of CSRD is 3 nm. For the RSR nanostructure, the nanorod length *a* is chosen as 180 nm for optimal match between nanorod and nanoring, other parameters are same as the RCSRD. In the scattering spectra of these two nanostructures, the quadrupolar Fano resonances are at 0.737 eV and 1.628 eV, respectively. Fano linewidth of RCSRD is 0.0187 eV, which is only 1/13 of the RSR nanostructure. There is a resonance peak at 1.71 eV in the scattering spectra of RCSRD, which comes mainly from the quadrupolar resonance of the CSRD. In the scattering spectra of RSR, there is a strong bonding mode at 0.86 eV, which comes from the primitive plasmon resonance of the rod and the ring. In Figure 2, this resonance peak is reduced to one fifth in order to show clearly the Fano resonances.

**Figure 3.**
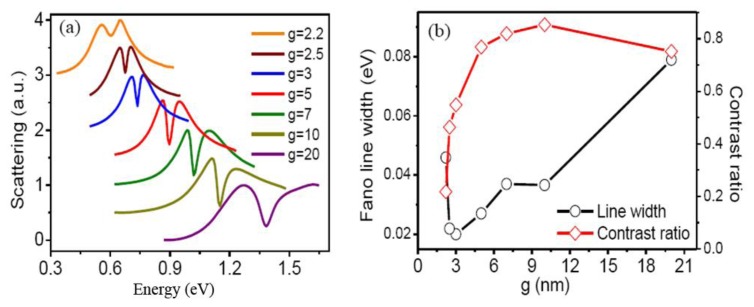
(**a**) The scattering spectra of RCSRD with different gap width *g*. As *g* decreasing from 20 to 2.2 nm, the corresponding nanorod lengths are of 180, 200, 250, 340, 480, 540 and 630 nm, respectively; (**b**) The Fano linewidth and contrast ratio as a function of the gap width *g*.

Figure 3a shows the influences of the nanogap *g* between the nanoring and the inner nanodisk on the quadrupolar Fano resonance. As the gap widths decrease from 20 to 2.2 nm, the Fano resonance peak gradually red shifts from 1.383 to 0.605 eV. However, Fano linewidth decreases greatly from 0.079 to 0.0187 eV when the gap decreases to 3 nm, and it increases abruptly to 0.0459 eV as the gap further decreases to 2.2 nm. The contrast ratio of Fano resonance increases gradually to 85%, and then decreases greatly to 22% as the gap decreasing from 20 to 2.2 nm, as shown in Figure 3b.

**Figure 4.**
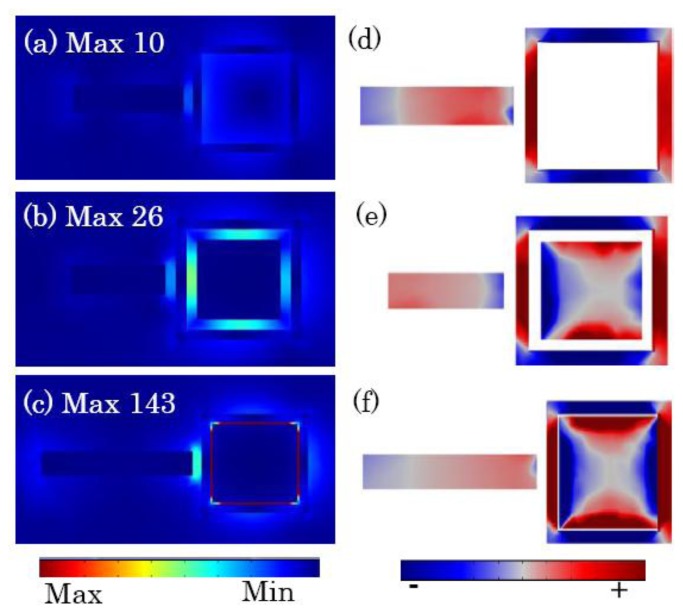
(**a**–**c**) The electric field amplitude in the middle section; (**d**–**e**) the induced surface charges on the top surfaces of the RCSRD with the gap *g* between the ring and disk of 100 (RSR), 20 and 5 nm, respectively.

The combination of the primitive dipole resonance of the outer nanorod and the antibonding plasmon resonance of CSRD forms the broad bright mode. It interacts with the narrow dark quadrupolar mode of the square nanoring, and induces the formation of the quadrupolar Fano resonance dip in the scattering spectra, as shown in Figure 3a. Figure 4 shows the electric field distribution and the induced surface charges on the top surfaces at the quadrupole Fano dips. The dark quadrupolar mode of the RCSRD are excited strongly, and the dipolar mode of the outside rod is suppressed greatly. If the CSRD structure has no the central disk, the maximum electric field localizes in the gap between the outside rod and the CSRD structure, which can enhance it 10 times. However, when the ring becomes a CSRD structure, the maximum electric field localizes in the gap in CSRD. When the gap *g* is 5 nm, the maximum electric field enhances to 143 times, as shown in Figure 4c. The charge distribution of the outside nanorod in Figure 4d,e demonstrates that the dipolar mode is further suppressed as the inner nanodisk in CSRD becomes larger.

**Figure 5.**
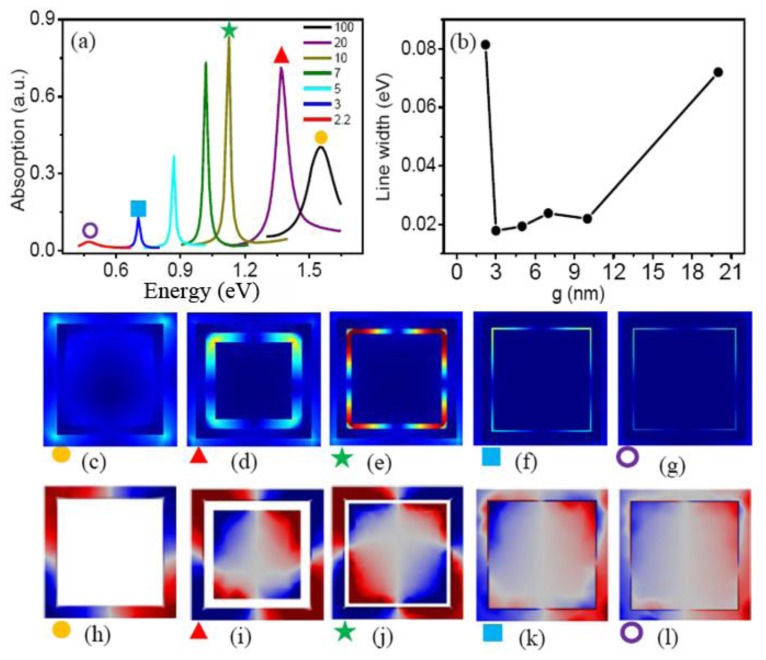
(**a**) The absorption spectra of the CSRD with different gap width *g*; (**b**) The line widths of Fano dips as a function of the gap width *g*; (**c**–**g**) The electric field distribution in the middle section and (**h**–**l**) the induced surface charges on the top surfaces at the quadrupolar resonance peaks in (**a**).

In order to understand the variation of line width and contrast ratio of the quadrupolar Fano resonances, we investigate the dark quadrupolar mode of the CSRD nanostructure. Figure 5a shows the absorption spectra of CSRD with different gap width *g*, which is excited by horizontal incident light. If the light incident along the vertical direction, they cannot be excited. They act as the dark modes of the quadrupolar Fano resonances. The resonance peak positions red shift as the nanogap width decreases, which is caused by two reasons: the coupling of the inner nanodisk and nanoring becomes stronger and the central length of nanocavity becomes longer.

Figure 5a shows that the intensity of the dark quadrupolar resonance increases when the gap *g* decreases from 100 to 10 nm, and then decreases as the gap decreases to 2.2 nm, which accords with the variation of the Fano contrast ratio of the RCSRD. Similarly, the resonance line width of the dark quadrupolar mode decreases as the gap *g* decreasing from 100 to 3 nm, and then increases greatly when the gap decreases to 2.2 nm, as shown in Figure 5b, which agrees with the linewidth of the Fano resonance of the RCSRD. In Figure 5a, the dark quadrupolar resonance shifts from 1.55 eV to 0.70 eV when the gap *g* decreases to 3 nm, which also accords well with the variation of the Fano resonance of the RCSRD in Figure 2. These results indicate that the strong red shift of the Fano resonance results from the coupling of the inner nanodisk and nanoring in CSRD [13]. Therefore, the dark quadrupolar mode of the CSRD nanostructure determines the Fano resonance of the RCSRD.

Figure 5c–g show the electric field distribution at the quadrupolar resonance peaks. If there is no the inner disk, the electric field distributes mainly out of the ring, as shown in Figure 5c. However, when a disk is put in the ring, the electric field intensity localizes strongly in the cavity because of the localized surface plasmon (LSP), as shown in Figure 5d,e. The line width decreasing originates from this LSP because it restrains the radiative damping. The localized surface plasmon results in the intensifying of the dark quadrupolar resonance as the gap *g* decreases to 10 nm. However, as the gap is less than 10 nm, the CSRD supports a hybrid mode (as shown in Figure 5k) consisted of a subradiant quadrupolar mode and a superradiant dipolar mode, so the intensity of the quadrupolar mode decreases. However, if the width *g* decreases to 2.2 nm, the subradiant quadrupolar mode changes to a superradiant dipolar mode, as shown by the surface charge distribution in Figure 5l. The strong radiative damping results in a drastic increase in the line width.

**Figure 6.**
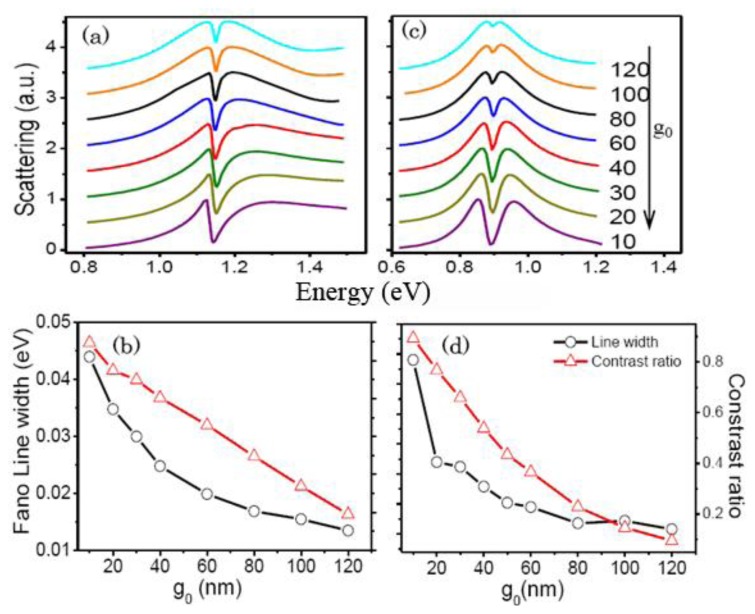
(**a**) The scattering spectra of the RCSRD and (**b**) the Fano linewidth and contrast ratio as functions of the gap width *g_0_* for the gap *g* of 10 nm and the rod length *a* of 145 nm; (**c**–**d**) The same information for the *g* of 5 nm and the rod length *a* of 340 nm.

Figure 6 presents the influence of the nanogap *g_0_* between the outer nanorod and the square nanoring on the quadrupolar Fano resonances. The Fano linewidth declines exponentially and the contrast ratio reduces gradually as *g_0_* increases. The gap width *g_0_* decides the coupling strength between the broad bright mode and the narrow dark mode. As *g_0_* increasing, the coupling strength becomes weaker. As a result, the Fano dip becomes narrower and shallower, which accords with the results in [5].

**Figure 7.**
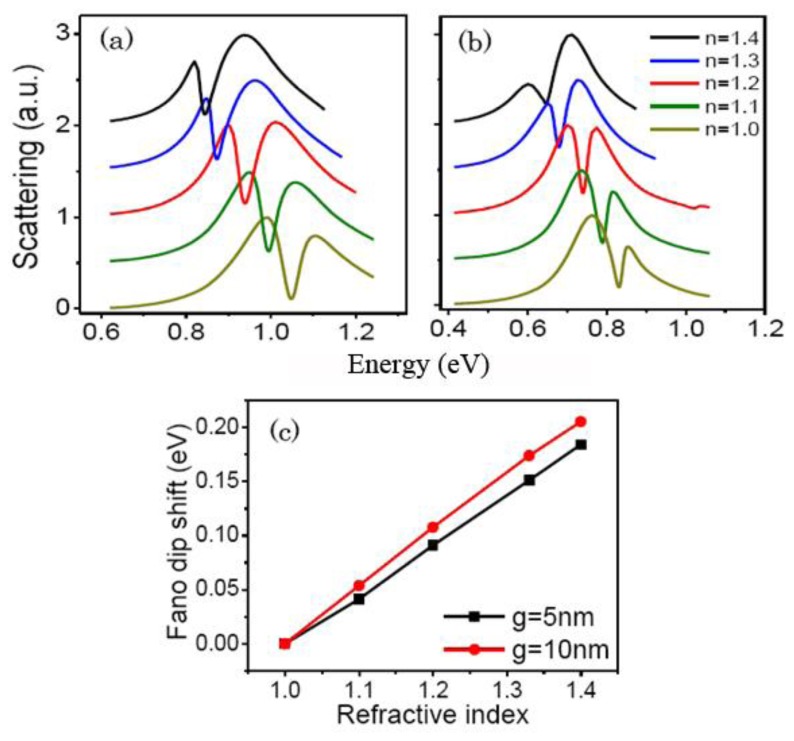
Scattering spectra of the RCSRD on a glass substrate surrounded by media with varying refractive indices for the gap *g* of 10 nm (**a**) and of 5 nm (**b**); (**c**) The Fano dip shifts with different refractive indices of surrounding dielectric environments.

Metamaterials with the sharp plasmon resonances have broad practical applications such as active plasmonic switching, slow-light optical devices, SERS, and sensing [29–32]. The RCSRD nanostructure can be employed as a tunable refractive-index based sensor because its resonance position depends on the dielectric constants of the surrounding media. To investigate the sensing performance, we calculate the scattering spectra with different dielectric environments, as shown in Figure 7. FOM is defined as the ratio of the sensitivity of surrounding medium to the linewidth of the Fano dip [33]. The RCSRD nanostructure is deposited on a glass substrate (*n* = 1.5) with a thickness of 60 nm. The ring nanogap *g* and the nanorod length *a* are fixed at 10 and 220 nm for Figure 7a, and are of 5 and 380 nm for Figure 7b. Figure 7 shows the scattering spectra of the RCSRD surrounded by different media with refractive indices *n* of 1, 1.1, 1.2, 1.33, and 1.4. The Fano dips red shift significantly with increasing refractive index. The sensitivities of the quadrupolar Fano resonances of these two structures are 0.461 and 0.513 eV/refractive index unit (RIU), and the Fano linewidths are 0.032 and 0.043 eV. The calculated FOMs are 15 and 12, respectively. However, the FOM of the quadrupolar mode of RSR nanostructure, namely, no inner disk in the RCSRD, is only 1.5. The FOM is enhanced 10 times. Therefore, the cavity mode is an effective way to decrease Fano linewidth and increase the FOM.

## 4. Discussion

**Figure 8.**
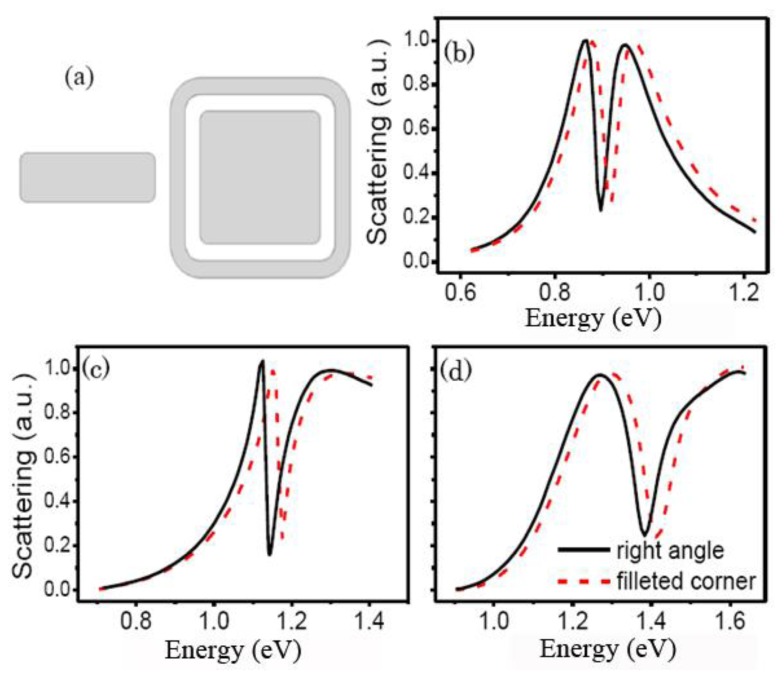
(**a**) Sketch of the RCSRD with filleted corner; (**b**–**d**) Scattering spectra of the RCSRD with the gap *g* of 5 nm, 10 nm and 20 nm, respectively.

The right angle in the RCSRD in Figure 1 is very difficult to fabricate. As the RCSRD nanostructure changes from a right angle to a filleted corner, as shown in Figure 8a, the Fano dip blue shifts slightly as the cavity length becomes shorter. However, the Fano linewidth of the quadrupolar Fano resonance is nearly unchanged.

**Figure 9.**
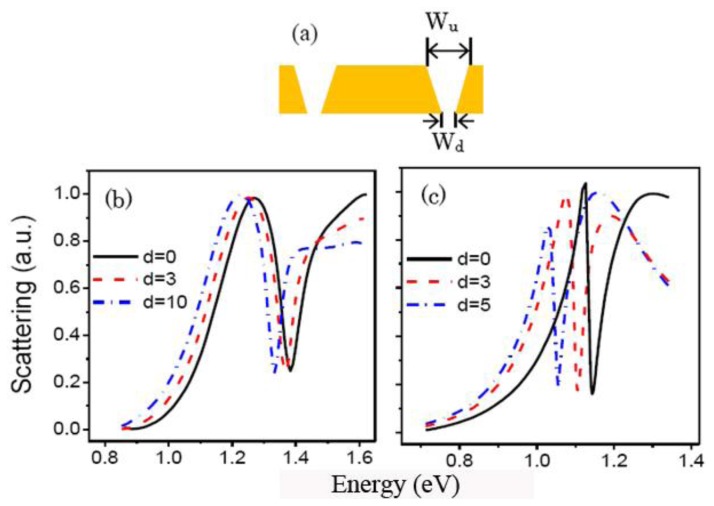
(**a**) Cross section of the CSRD nanostructure with wedge-shaped gap; (**b**,**c**) Scattering spectra of the RCSRD with *W_u_* of 20 nm and 10 nm, respectively.

The morphosculpture of the narrow and deep gap in actual experiments is usually wedge-shaped. The top width of the gap is noted as *W_u_*, the bottom width of the gap is *W_d_*. The difference is *d* = *W_u_* − *W_d_*, as shown in Figure 9a. We investigate the influence of wedge-shaped gap on the quadrupolar Fano resonance. Figure 9b,c shows the scattering spectra of the RCSRD with the change of *d*. When the top width *W_u_* is 20 nm, the quadrupolar Fano dip position red shifts from 1.38 to 1.33 eV, the line width decreases from 0.079 to 0.051 eV as *d* increases from 0 to 10 nm. In this case, in order to obtain the Fano dip we need, we can decrease slightly the size of the RCSRD nanostructure.

**Figure 10.**
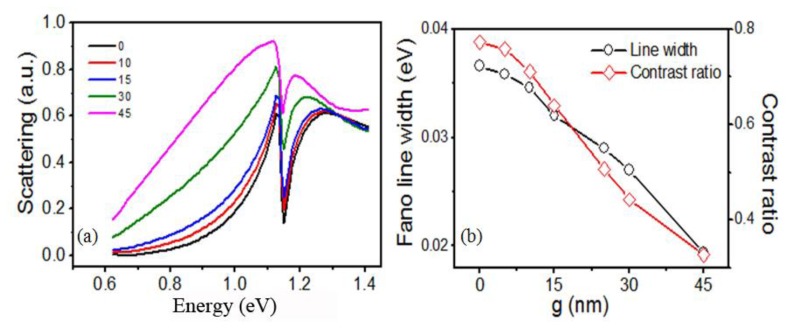
(**a**) The scattering spectra of the RCSRD and (**b**) the Fano linewidth and contrast ratio as a function of the incident angle *θ*. The geometric parameters are: the gap *g* = 10 nm, *g*_0_ = 20 nm, and the rod length *a* = 145 nm.

Figure 10 presents the scattering spectra of the RCSRD as a function of the incident angle *θ*. The incident angle *θ* is defined as the angle between the incident light and the normal direction of the sample. The Fano resonance peak position does not move as *θ* increases from 0° to 30°. The Fano linewidth and the contrast ratio reduced only 5.4% and 7.9% as the incident angle *θ* increases from 0° to 10°. As the angle *θ* is larger than 10°, they decrease greatly.

The scattering spectra above are the total scattering spectrum, but they are not directly measurable. We calculate the forward or backward scattering spectra, and find they are same completely. Compared with the total scattering spectra, the Fano linewidth and the contrast ratio are almost same, only the spectra intensity decreases by a half.

## 5. Conclusions

In summary, we have proposed a metallic nanostructure consisting of a concentric square ring-disk nanostructure and an outside nanorod. The quadrupolar dark mode of CSRD becomes narrow and intense as the gap between ring and disk decreases because of the localized surface plasmon of the cavity in CSRD, and it determines mainly the Fano line shape in the RCSRD nanostructure. Fano linewidth and spectral contrast ratio can be actively manipulated by adjusting the gap between the nanorod and CSRD, and by adjusting the gap between the ring and disk in CSRD. As the gap in CSRD is reduced to 5 nm, the quadrupolar Fano resonance is only 0.025 eV wide with a contrast ratio of 80%. When the RCSRD nanostructure is deposited on a glass substrate, the FOM reaches to 15, which is 10 times higher than that without the inner disk in CSRD. This RCSRD nanostructure has potential applications as a sensitive sensor.
